# Morbidity and mortality of patients with peritoneal malignancy following cytoreductive surgery and hyperthermic intraperitoneal chemotherapy

**DOI:** 10.1007/s12672-024-00968-4

**Published:** 2024-04-05

**Authors:** Greta Hotza, Michael Karageorgos, Varvara Pastourmatzi, Nader Baniowda, Dimitrios Kyziridis, Apostolos Kalakonas, Nicolaos Chavouzis, Irene Hotza, Antonios-Apostolos Tentes

**Affiliations:** 1 Department of Anesthesiology, EUROMEDICA Kyanous Stavros, Viziis 1, 54636 Thessaloniki, Greece; 2 Intensive Care Unit, EUROMEDICA Kyanous Stavros, Viziis 1, 54636 Thessaloniki, Greece; 3 Department of Surgical Oncology, Peritoneal Surface Malignancy Program, EUROMEDICA Kyanous Stavros, Viziis 1, 54636 Thessaloniki, Greece

**Keywords:** Morbidity, In-hospital mortality, Cytoreductive surgery, HIPEC

## Abstract

**Background:**

The purpose of this study was to record the incidence, and identify the prognostic variables of morbidity and mortality in patients with peritoneal malignancy undergoing cytoreductive surgery (CRS) combined with hyperthermic intraperitoneal chemotherapy (HIPEC).

**Methods:**

The files of patients with peritoneal malignancy who underwent CRS + HIPEC from 2015–2022 were retrieved. Morbidity and hospital mortality were recorded and correlated to a variety of clinical variables.

**Results:**

A total of 44/192 (22.9%) patients were recorded with postoperative complications. Grade 3 and 4 complications were 12.5%. The possible prognostic variables of morbidity were the extent of peritoneal malignancy and the number of suture lines. The mortality rate was 2.5% (5 patients). The number of FFP units, and peritonectomy procedures were identified as possible prognostic variables of hospital mortality.

**Conclusions:**

The morbidity rate in patients undergoing CRS + HIPEC is acceptable compared to morbidity of previous publications or major gastrointestinal surgical operations. The possible prognostic variables of morbidity are the extent of peritoneal malignancy, and the number of suture lines. The mortality rate is low. The possible prognostic variables of mortality are the number of FFP units, and the number of peritonectomy procedures.

## Introduction

Cytoreductive surgery (CRS) combined with hyperthermic intraperitoneal chemotherapy (HIPEC) is considered the standard treatment in selected patients with peritoneal malignancy. The purpose of the surgical procedure is the removal of all or nearly all macroscopically visible tumors, while the purpose of HIPEC is the eradication of microscopic residual tumor [1]. The combination of CRS and HIPEC has improved the long-term survival of patients with peritoneal malignancy. Survival depends on the biological behavior of the primary tumor, the patient’s performance status, the completeness of cytoreduction, and the extent of peritoneal disease [2]. Before the introduction of cytoreductive surgery, peritoneal malignancy was considered an inoperable disease, and these patients were treated with systemic chemotherapy without any chance to survive more than 9–12 months. Over the last 40 years, CRS in combination with HIPEC has been extensively used in the treatment of peritoneal malignancy. Improved survival [3] has been recorded, particularly in patients with colorectal cancer and peritoneal carcinomatosis [4], pseudomyxoma peritonei [5], peritoneal mesothelioma [6], locally advanced ovarian cancer [7], and other diseases with peritoneal carcinomatosis [8, 9]. Patients with peritoneal malignancy usually need to undergo extensive surgical resections, which include standard peritonectomy procedures and other additional visceral resections [10]. Older publications report a high morbidity rate varying from 39 to 67.6% and a high mortality rate from 7.6 to 9% [11–13]. In 1996, Sugarbaker pioneered CRS and HIPEC and reported a mortality rate of 5% and a morbidity of 35% [14], which were reduced to 1.5% and 27%, respectively, in a few years [15]. In 2006, the morbidity rate remained at 2% and the mortality at 40% [16]. The Dutch and Australian experiences were similar, pointing out that the time the experience increased, the morbidity and mortality decreased [17, 18]. It appears that teams undertaking the method achieve the “global learning curve” and reach a plateau. As a consequence, if the learning curve is not used, the morbidity and mortality are expected to be unacceptably high.

The study was designed to assess the incidence and identify the possible prognostic variables of morbidity and in-hospital mortality in one tertiary center for peritoneal malignancy (EUROMEDICA Kyanous Stavros) from the same surgical and anesthesiological teams in which the learning curve had long ago been achieved. Hopefully, the surgical and the anesthesiological team, using the results of morbidity and mortality will improve the results in the future by avoiding possible mistakes.

## Patients-methods

The database of patients with peritoneal malignancy was prospectively maintained from November 2015 until December 2022 in one center (Department of Surgical Oncology, EUROMEDICA Kyanous Stavros, Thessaloniki, Greece). During this period, patients with peritoneal malignancy of various primaries who underwent extensive CRS (standard peritonectomy procedure, and visceral resections) in combination with HIPEC were included in the study.

Patients with open-close procedures, those who underwent CRS without HIPEC, those who underwent minor surgery such as reconstruction of a stoma following extensive surgery, and those who underwent HIPEC for palliative reasons such as malignant ascites were not included in the study. In addition, approximately 800 patients who underwent CRS and HIPEC by the same surgical team in other hospitals from January 2003 until December 2015 were not included in the study. The patients’ age, the gender, the performance status, the classification according to ASA scale, the tumor volume, the tumor grade, the extent of previous surgery (PSS), the extent and distribution of peritoneal malignancy (PCI), the completeness of cytoreduction (CC-score), the number of peritonectomy procedures, the number of anastomoses, the units of transfused blood during surgery, the units of transfused fresh frozen plasma (FFP) during surgery, the duration of surgery, and the primary site were all recorded in detail.

The details of the previous surgical report made it possible to assess the prior surgery score (PSS) [19]. The medical history and the physical examination made it possible to assess the patient according to ASA classification. The physical examination of the patient by both the anesthesiologist, and the surgeon made possible the assessment according to the Karnofsky performance scale.

### Surgical management

All patients underwent mechanical bowel preparation the day before surgery. An epidural catheter was placed just before-intubation for postoperative abdominal pain control when the patient was still awake. One central line (either subclavian or intrajugular), one peripheral venous line, one transcutaneous arterial line, a Folley catheter, and a gastrointestinal tube were always placed as soon as the patient was intubated. The patients received piperacillin plus tazobactam 4.5 g every 6 h and metronidazole 500 mg every 8 h. Antibiotics were administered just before the surgical incision and continued for 5 days. Modification of antibiotic administration was possible in case of infection according to cultures. Antithrombotic stockings and administration of low molecular weight heparin (LMWH) derivatives were routinely used and discontinued after the 20th postoperative day. Post-splenectomy vaccination was always ordered if splenectomy was performed. After the completion of the operation, one suction-drain was placed under the right hemidiaphragm, another under the left hemidiaphragm, and another at the right subhepatic space whenever cholecystectomy was performed, and two more drains were placed at the pelvis. A thoracostomy tube was always put in place to drain the pleural cavity for at least 5 days whenever a subdiaphragmatic peritonectomy procedure was performed for protection of postoperative pleural effusion. The epidural catheter was left in place for 2–4 days postoperatively. The gastrointestinal tube was removed as soon as the bowel movement returned and the anastomoses were considered safe. The central line was usually removed around the 7th–10th postoperative day. After the 10th day, the site of the central line was always routinely changed even if signs of infection were not present. All patients were extubated in the ICU, where they remained for 24–48 h until complete hemodynamic stabilization.

A midline abdominal incision extending from the xiphoid process to the symphysis pubis was always used for maximal exposure of the abdominal cavity. The tumor volume and the extent of peritoneal malignancy were assessed after complete lysis of the adhesions, which made it possible calculate the peritoneal cancer index (PCI) [20]. Patients with small-volume tumors were considered those that were found to have nodules with a maximal diameter < 0.5 cm, while those with a maximal diameter > 0.5 cm or those with confluent nodules of any size were considered large-volume tumor patients [20]. At the end of surgery, the number of peritonectomy procedures was recorded, and the completeness of the cytoreduction score was assessed [19]. The peritonectomy procedures are as follows: epigastric peritonectomy (resection of round and falciform hepatic ligaments with the old scar and the umbilicus), right and left subdiaphragmatic peritonectomy procedure, greater omentectomy with or without splenectomy, lesser omentectomy, right and left lateral peritonectomy, cholecystectomy with or without resection of the omental bursa, and pelvic peritonectomy procedure [2]. The epigastric peritonectomy procedure was used in reoperations. Cholecystectomy was routinely performed even if there were no implants on its surface. Visceral resections except those included in standard peritonectomy procedures are considered separately as additional procedures. These are as follows: right colectomy, subtotal colectomy, segmental intestinal resection, subtotal or total gastrectomy, subtotal or total pancreatoduodenectomy, and distal pancreatectomy.

After tumor resection and before the reconstruction of the alimentary tract, HIPEC was performed for 60–90 min (depending on the cytostatic drug that was used) at 42–43 °C. HIPEC was administered using the open abdominal (Coliseum) technique. The skin edges of the abdominal cavity were adequately elevated so that 2–3 L of prime solution could be instilled. A heater circulator with two roller pumps, one heat exchanger, one reservoir, an extracorporeal system of two inflow and two outflow tubes, and 4 thermal probes was used for HIPEC (Sun Chip, Gamida Tech, France). A prime solution of 2–3 L of normal saline or Ringer’s lactate was instilled prior to the administration of the cytostatic drug, and as soon as the mean abdominal temperature reached 40 °C, the cytostatic drugs were instilled in the abdomen.

The reconstruction of the continuity of the gastrointestinal tract was performed after the completion of HIPEC. Proximal stoma defunctioning was always performed in those cases in which more than two anastomoses needed to be protected.

Mit-C (15 mg/m^2^) in combination with doxorubicin (15 mg/m^2^) was used for 90 min in HIPEC for gastrointestinal malignancies in addition to 5-FU (400 mg/m^2^) plus leucovorin (20 mg/m^2^) injected IV. Cisplatin (50 mg/m^2^) combined with doxorubicin (15 mg/m^2^) was used for 90 min in HIPEC for gynecologic malignancies, peritoneal mesothelioma, and peritoneal sarcomatosis in addition to ifosphamide (1300 mg/m^2^), and mesna (260 mg/m^2^), which were administered IV. Gemcitabine (1000 mg/m^2^) was used for 60 min in HIPEC for pancreatic carcinomas. In a few cases of pancreatic cancer, the patients received 5-FU (400 mg/m^2^) plus leucovorin (20 mg/m^2^) intravenously.

All specimens were histologically examined, and the tumor grade was identified. The histological type of the tumor, the depth of invasion of other resected organs, and the site and infiltration of the resected lymph nodes were recorded in addition to other histopathologic details [21].

The study was approved by the Ethical Committee of the Hospital (EUROMEDICA Kyanous Stavros Scientific Committee), and all patients signed an informed consent form.

### Statistical analysis

Statistical analysis was possible using SPSS (Statistical Package for Social Sciences, version 17). All recorded variables were correlated with morbidity and mortality. Continuous variables were analyzed by using Student’s t-test. Fisher’s exact test was used to analyze categorical variables. Logistic regression analysis was used in a multivariate analysis to determine the prognostic variables for morbidity and hospital mortality. The backward elimination method was used to determine which clinical variables best predicted the presence of morbidity and hospital mortality. A P value < 0.05 was considered statistically significant.

## Results

The files of 192 patients who underwent CRS and HIPEC in EUROMEDICA Kyanous Stavros were retrospectively retrieved and reviewed. There were 43 (22.4%) men and 149 (77.6%) women. The mean age of the patients was 56.5 ± 12.1 (24–83) years. The mean PCI was 11.4 ± 7.7 (1–33). The primary site of the peritoneal malignancy is listed in Table 1. As expected, the majority of patients were women with ovarian cancer, which is the most frequent disease presenting peritoneal metastases. The clinical characteristics of the patients are listed in detail in Table 2. The patients were carefully selected, and the vast majority of them were found to have acceptable performance status, without major comorbidities, and had large volume tumors. Complete cytoreduction was possible in the majority of patients, although half of them had undergone extensive surgery in the past. In 19 patients (9.9%) at the end of the surgical procedure, a loop-ileostomy was considered mandatory because more than two anastomoses distal to the ileostomy should be protected. The ileostomy was frequently reconstructed 4–6 weeks after the initial operation. A total of 44 (22.9%) patients demonstrated at least one complication (Table 3). According to the Clavien–Dindo classification, there were 6 patients (3.1%) with grade I complications, 9 (4.7%) with grade II complications, 4 (2.1%) with grade III complicatio0ns, and 20 (10.4%) with grade IV complications. Univariate analysis revealed that the extent of peritoneal malignancy (PCI), the number of anastomoses and the number of FFP units were related to morbidity (Table 4). However, the possible prognostic variables of morbidity were the PCI (p = 0.012), and the number of anastomoses (p = 0.002) (Table 4).

**Table 1:** Primary site of peritoneal malignancy in 192 patients

Primary site	Ν	%
Ovaries	102	53.1
Large bowel	26	13.5
Appendix	18	9.4
Stomach	11	6
Pancreas	10	5.8
Peritoneal mesothelioma	11	6
Corpus uteri	7	3.1
Peritoneal sarcomatosis	7	3.1

**Table 2:** Clinical characteristics

Variable	Ν	%
ASA stage		
I	178	92.7
II	14	7.3
Performance status		
90–100%	186	96.9
70–80%	6	3.1
Tumor volume		
Large volume	174	90.6
Small volume	18	9.4
Completeness of cytoreduction score		
CC-0	132	68.8
CC-1	60	31.3
Prior surgery score		
PSS-0	64	33.3
PSS-1	33	172
PSS-2	72	37.5
PSS-3	23	12
No of peritonectomy procedures	6 ± 3 (1–12)	
No of anastomoses	2 ± 1 (0–5)	
Blood units	1 ± 1 (0–4)	
FFP units	2 ± 2 (0–8)	
Duration of hospitalization	13 ± 8 (1–76)

**Table 3:** Complications in 44 (22.9%) patients

Complication	Ν	%
Pulmonary embolism	1	0.5
Pneumonia	3	1.6
Cardiac arrhythmias	4	2.1
Acute renal failure	1	0.5
Cerebrovascular accident	1	0.5
Postoperative bleeding	3	1.5
Anastomotic failure	4	2.1
Central line sepsis	1	0.5
Wound infection	5	2.6
Upper gastrointestinal tract bleeding	2	1
Pleural effusion	4	2.1
Grade 4 hematologic toxicity	2	1
Pneumothorax	1	0.5
Urine leak	2	1
Bile leak	1	0.5
Paralytic ileus	1	0.5
Peripancreatitis	1	0.5
Intra-abdominal abscess	5	2.6
Enterocutaneous fistula	2	1

**Table 4:** Morbidity

Univariate analysis		Multivariate analysis		
Variable	P value	P value	HR	95% CI
ASA stage	0.318			
Performance status	0.85			
Gender	0.218			
Tumor volume	0.05			
Tumor grade	0.338			
CC-score	0.256			
PSS	0.813			
Age > 65 years	0.118			
PCI	0.003	0.012	6.374	1.143–2.896
Primary site	0.069			
No of anastomoses	0.008	0.002	9.375	1.236–2.624
Units of transfused blood	0.155			
Units of transfused FFP	0.035			
Duration of surgery	0.187			
No of peritonectomy procedures	0.367			

The 30-day in-hospital mortality (Grade V complications) was 2.5% (5 patients). One patient died because of cerebrovascular accident, another because of acute renal failure, another because of postoperative bleeding that was successfully managed but later developed sepsis, and a fourth patient died of intra-abdominal abscess, although it was adequately drained. The fifth patient was given a high dose of heparin because of pulmonary embolism and died of uncontrollable intra-abdominal haemorrhage. Univariate analysis revealed that the FFP units, and the number of peritonectomy procedures were related to mortality (Table 5). The number of FFP units (p < 0.001), and the number of peritonectomy procedures (p = 0.024) were identified as possible prognostic indicators of hospital mortality (Table 5).

**Table 5:** 30-day in-hospital mortality

Univariate analysis		Multivariate analysis		
Variable	P value	P value	HR	95% CI
ASA stage	0.318			
Performance status	0.921			
Gender	0.312			
Tumor volume	1			
Tumor grade	0.303			
CC-score	0.177			
PSS	0.448			
Age > 65 years	0.595			
PCI	0.019			
Primary site	0.901			
Units of transfused blood	0.053			
Units of transfused FFP	< 0.001	< 0.001	1.032	0.007–4.796
Duration of surgery	0.067			
No of peritonectomy procedures	< 0.001	0.024	5.109	0.124–0.862
No of anastomoses	0.001			

## Discussion

Surgical removal of the tumor load by using CRS combined with HIPEC is considered the most powerful therapeutic tool in the treatment of peritoneal malignancy [6, 8, 22]. The role of HIPEC in colorectal cancer with peritoneal metastases was strongly questioned recently [23], although it was considered equally effective to surgery in the past. However, CRS alone or in combination with HIPEC is considered a therapeutic modality that is associated with morbidity and mortality which is not very different from a major gastrointestinal surgical procedure, such as Whipple’s procedure [24, 25]. The unacceptable short-term outcomes [11, 12] that have been reported in the past probably reflect the center’s inexperience or improper patient selection and have led to the decision that a learning curve must necessarily be achieved [18, 26–28]. The Basingstoke experience showed that morbidity and mortality decreased significantly as soon as the experience increased. The anastomotic failures fell from 12 to 0% with the routine use of proximal stomal defunctioning. Reoperations for postoperative bleeding fell from 11 to 0% with meticulous hemostasis. Another significant factor responsible for the reduction in morbidity and mortality was proper patient selection [27], which is in agreement with the Dutch experience [17, 29]. International practice has confirmed that proper patient selection and adequate experience achieved by the completion of the learning curve has indeed contributed to the decrease in morbidity and mortality in patients undergoing CRS and HIPEC [27]. The risk of severe and probably fatal complications is very high in patients with large-volume tumors and high-grade disease who cannot undergo complete or near complete cytoreduction.

In our study, the morbidity rate of any grade was 22.9%, and was related to the extent of peritoneal disease, the number of anastomoses, and the number of FFP units. However, only the number of anastomoses and the extent of peritoneal disease were recorded as possible prognostic variables of morbidity. Severe morbidity (Grade III and IV), which included postoperative bleeding, anastomotic failures, upper gastrointestinal tract bleeding, intra-abdominal abscess, grade 4 hematologic toxicity, cerebrovascular accident, acute renal failure, urine and bile leak, and pulmonary embolism requiring either reoperation or ICU stay or any other surgical or radiological intervention was limited to 12.5%.

One relatively recent publication showed that the prognostic variables for severe morbidity (Grade 3 and 4) were extensive previous surgery, recent smoking history, poor performance status, and extensive cytoreduction [30]. One of the first publications showed that morbidity was related to the duration of surgery, the number of peritonectomy procedures, and the number of suture lines [15]. In another more recent multicentric publication, enterocutaneous fistulas and anastomotic leaks were found to be the most frequent complications. Extensive lysis of fibrous or cancerous adhesions results in seromuscular tears of the bowel that later develop into fistulas [16]. Bowel obstruction, prior intraperitoneal chemotherapy, and/or radiotherapy have been identified as additional causes of fistula formation [16]. The administration of HIPEC is of crucial importance and has an adverse effect on wound healing, making fistula formation and anastomotic failures more probable. A difficult splenectomy in which the pancreatic capsule is disrupted usually leads to postoperative peripancreatitis [15]. Pancreatic or rectal resections, and multiple anastomoses are related to high morbidity, while age > 70 years, and reoperation are related to hospital mortality [30]. Distal pancreatectomy is frequently associated with pancreatic leaks that result in intra-abdominal abscess [30]. In tertiary high-volume centers, the rate of grade III/IV morbidity varied from 12–67.6%, and hospital mortality varied from 0.9–9% [27]. The most frequent postoperative complications included sepsis, fistulas, abscess, prolonged ileus, perforation, anastomotic leak, deep venous thrombosis or pulmonary embolization, hematologic toxicity, and renal failure [27].

Postoperative hemorrhage was limited to 1.5% in our study because of meticulous hemostasis. Anastomotic failure was recorded in 2.1% of patients. Proximal stomal formation was not routinely performed unless there were more than two distal anastomoses that should be protected or if the anastomoses did not seem to be safe. Although proton pump inhibitors (PPIs) are routinely given to all patients undergoing CRS and HIPEC, upper gastrointestinal tract bleeding was recorded in 1% of patients and was attributed to low molecular weight heparin (LMWH) derivatives that are routinely administered postoperatively. Peripancreatitis was limited to 0.5% and fistulas to 1%, although approximately half of the patients had undergone extensive surgery in the past and required extensive lysis of the adhesions. In contrast to other publications haematologic toxicity was recorded in only 1% of the complicated patients despite the use of simultaneous intravenous chemotherapy with HIPEC [18, 29]. Such a low incidence of hematologic toxicity is attributed to low doses of cytostatic drugs. The low rate of postoperative pleural effusion is attributed to the routine use of pleural drains following subdiaphragmatic peritonectomy procedures. Central line sepsis was recorded in 0.5% because usually the venous catheters are removed or changed at the 7th to 10th postoperative day. Prolonged bowel paralysis was recorded in one patient who underwent extensive surgical manipulation (resection or elecauterization of implants) at the mesentery of the small bowel.

The incidence of 30-day in-hospital mortality was 2.5%. One patient died from uncontrollable intra-abdominal haemorrhage, and the others ultimately died of multiple system organ failure (MSOF) despite the initial fatal complication. The number of FFP units, and the number of peritonectomy procedures were recorded as the possible prognostic variables of hospital mortality. The majority of patients were classified as ASA stage I, and II, and their performance status was more than 70%. Therefore, ASA stage and performance status were not related to either morbidity or mortality. Advanced age was not found to be related to either morbidity or mortality.

The international literature has provided enough evidence that by the time the experience increases, the incidence of morbidity and mortality is decreased. In fact, the magic number of at least 110 [28] or according to others 140 to 150 cytoreductions [18, 26] is considered the required step for the achievement of the learning curve. In addition, morbidity and mortality may be acceptable if candidates for CRS and HIPEC must be restricted to those patients who may undergo complete or near-complete cytoreduction [17, 18, 24, 26, 27].

The major advantage of the study is that all patients underwent surgery by the same surgical and anaesthesiological team. Although this is a retrospective study, the database was prospectively maintained. This definitely confirms that if the learning curve is achieved, CRS and HIPEC may be safely performed, and the incidence of morbidity and mortality may be low and acceptable.

## Conclusions

CRS in combination with HIPEC has been established as the most effective treatment in patients with peritoneal malignancy. The morbidity rate in patients undergoing CRS + HIPEC is acceptable and comparable to previous studies’ morbidity or to major gastrointestinal surgery and may be recorded after the learning curve of the method has been achieved. The extent of peritoneal malignancy and the number of anastomoses have been identified as possible prognostic variables of morbidity. The mortality rate is low. The possible prognostic variables of mortality are the FFP units, and the number of peritonectomy procedures.

## Data Availability

The data that support the findings of this study are not openly available. The data are available from the corresponding author upon reasonable request.
